# Hong Kong Drug Allergy Delabelling Initiative (HK-DADI) consensus statements for penicillin allergy testing by nonallergists

**DOI:** 10.3389/falgy.2022.974138

**Published:** 2022-09-05

**Authors:** Philip H. Li, Jane C. Y. Wong, Jacky M. C. Chan, Thomas S. H. Chik, M. Y. Chu, Grace C. H. Ho, W. S. Leung, Timothy C. M. Li, Y. Y. Ng, Rocky Shum, Winnie W. Y. Sin, Eugene Y. K. Tso, Alan K. L. Wu, Elaine Y. L. Au

**Affiliations:** ^1^ Division of Rheumatology & Clinical Immunology, Department of Medicine, The University of Hong Kong, Queen Mary Hospital, Hong Kong SAR, China; ^2^ Division of Infectious Diseases, Department of Medicine & Geriatrics, Kwong Wah Hospital, Hong Kong SAR, China; ^3^ Division of Infectious Diseases, Department of Medicine, Queen Elizabeth Hospital, Hong Kong SAR, China; ^4^ Division of Rheumatology, Department of Medicine, Queen Elizabeth Hospital, Hong Kong SAR, China; ^5^ Division of Infectious Diseases, Department of Medicine & Therapeutics, Prince of Wales Hospital, Hong Kong SAR, China; ^6^ Division of Infectious Diseases, Department of Medicine & Geriatrics, Tuen Mun Hospital, Hong Kong SAR, China; ^7^ Division of Microbiology, Department of Clinical Pathology, Tuen Mun Hospital, Hong Kong SAR, China; ^8^ Division of Infectious Diseases, Department of Medicine, Alice Ho Miu Ling Nethersole Hospital, Hong Kong SAR, China; ^9^ Division of Infectious Diseases, Department of Medicine & Geriatrics, United Christian Hospital, Hong Kong SAR, China; ^10^ Division of Microbiology, Department of Clinical Pathology, Pamela Youde Nethersole Eastern Hospital, Hong Kong SAR, China; ^11^ Division of Clinical Immunology, Department of Pathology, Queen Mary Hospital, Hong Kong SAR, China

**Keywords:** allergy, consensus, Hong Kong, penicillin, nonallergist

## Abstract

**Introduction:**

Penicillin allergy testing has been traditionally performed by allergists, but there remains a huge deficit of specialists. A multidisciplinary effort with nonallergists would be invaluable to overcome the magnitude of penicillin allergy labels *via* the Hong Kong Drug Allergy Delabelling Initiative (HK-DADI). These consensus statements (CSs) offer recommendations and guidance to enable nonallergists to screen for low-risk (LR) patients and perform penicillin allergy testing.

**Methods:**

CSs were formulated by the HK-DADI Group using the Delphi method. An agreement was defined as greater than or equal to 80% consensus.

**Results:**

A total of 26 CSs reached consensus after multiple rounds of Delphi. CSs were categorized into risk assessment, skin testing, drug provocation testing (DPT), and post-testing management. For risk assessment, the essentials of allergy history and exclusion criteria were detailed. Patients with only LR features can proceed with testing by nonallergists. Skin tests should be performed prior to DPT. Details regarding the timing, preparation, and interpretation of skin tests were elaborated. DPT remains the gold standard to diagnose genuine allergy or tolerance and should be performed when there is a low pretest probability following negative skin testing. Details of DPT preparations, dosing protocols, and interpretation were elaborated. For post-testing management, inaccurate allergy labels should be delabeled following negative DPT with proper patient counseling.

**Conclusion:**

CSs support penicillin allergy testing by nonallergists in Hong Kong. LR cases can be managed by nonallergists at Spoke Clinics, with training and support of an allergist-led Hub.

## Introduction

*β*-lactam antibiotics (including penicillins, cephalosporins, carbapenems, and monobactams) are the most widely used but are most frequently associated with drug allergy (1). In Hong Kong, approximately 1 in 50 people have documented *β*-lactam “allergies,” and around 8,000 additional allergy labels are generated every year (2). However, many physicians and patients mistakenly report nonallergic adverse reactions as allergies, and almost 90% of labeled *β*-lactam allergies are found to be incorrect following a complete allergy workup (2, 3). Especially for penicillins, false allergy labels severely limit future antibiotic choices and are associated with a multitude of adverse clinical consequences, including the development of antimicrobial resistance (4–6). In Hong Kong, mislabeled penicillin allergies and their associated adverse outcomes are even higher among hospitalized and high-risk patients (7–9).

Penicillin allergy testing includes taking a comprehensive allergy history, followed by penicillin skin testing and, if negative, a penicillin provocation test (also known as a “challenge”). Traditionally, in Hong Kong, entire penicillin allergy testing has been performed by Specialists in Immunology and Allergy based on local experience adapted to the British Society for Allergy and Clinical Immunology (BSACI) standards (10). However, there is a huge deficit of allergy services and specialists in Hong Kong (11, 12). At the time of writing, the Hong Kong West Cluster (HKWC) remains the only center with a Specialist in Immunology and Allergy and formal penicillin allergy delabeling services (for adult patients) in the public sector. The current waiting time for a routine consultation at the HKWC specialist outpatient clinic is currently in excess of 8 years. Experience from other countries has shown that selected patients with suspected penicillin allergy can be delabeled successfully by clinicians who are not trained in allergy (i.e., nonallergists) (13–16). A multidisciplinary effort with nonallergists would, therefore, be extremely invaluable to overcome the magnitude of penicillin allergy labels.

In view of this, we propose a “Hub-and-Spoke” model—the Hong Kong Drug Allergy Delabelling Initiative (HK-DADI)—to be implemented to address the enormity of the penicillin allergy burden in Hong Kong. A similar model has proven to be successful in other multidisciplinary allergy initiatives (17). Allergists in the Hub will arrange formal training for all nonallergists in each respective center (“Spokes”) with in-person hands-on training, including risk stratification, conducting and interpreting skin tests, and post-testing management. Nonallergists will also undergo simulation training, assessed by allergists, to ensure they are confident with penicillin allergy testing. Patients with a penicillin allergy label may be triaged by nonallergists at their respective Spokes into “low risk” (LR) or “non-LR” (NLR). LR patients are deemed to be at (1) low risk of genuine penicillin allergy and/or (2) severe potential reactions, and can proceed with completing penicillin allergy workup by nonallergists. NLR patients can be adequately counseled and referred to Specialists in Immunology and Allergy for further workup (“Hub”). Regular training, support, and management of difficult cases at the Spokes would also be provided by the Hub. With the growing role of telemedicine, regular teleconferencing will be arranged for dynamic communication and enhancing opportunities for shared care between the Hub and Spokes.

In order to establish HK-DADI, this set of consensus statements (CSs) aims to offer clear recommendations and guidance to enable nonallergists to screen for LR patients and perform penicillin allergy testing. It also aims to provide a foundation and guide to set up LR allergy centers under the “Hub-and-Spoke” model.

## Methods

CSs were formulated using the Delphi method, which has been utilized to develop another allergy-related consensus in Hong Kong (18). Based on their experience in establishing prior allergy clinics and allergy-related CS, two facilitators (PHL and EYLA) were appointed from the HKWC to conceptualize and formulate the recommendations. An open call was made to all Departments of Medicine within the Hong Kong Hospital Authority for physicians with experience and/or interest in penicillin allergy delabeling to form the HK-DADI working group. A total of 13 physicians formed the group, comprising representatives from all seven hospital clusters. No honoraria were paid for participation.

In the first Delphi round, the voting group held a conference with a discussion on items warranted for penicillin allergy testing. The preliminary statements were then first construed by the two main facilitators, PHL and EYLA, with a range of different options available for each aspect of the CS. All members were also invited to suggest additional options if deemed necessary or more appropriate. During the second round of Delphi, all group members completed an online questionnaire to rate their agreement with each CS on a five-point Likert scale. Responses were graded as “Strongly Agree,” “Tend to Agree,” “Neither Agree nor Disagree,” “Tend to Disagree,” and “Strongly Disagree” for each respective statement scoring +1, +0.5, 0, −0.5, and −1, respectively. Scores were reported as a mean and standard deviation (SD). More extreme scores and lower SD indicated stronger consensus. The consensus was defined *a priori* as agreement by at least 80% of the respondents. In the third and final round of Delphi, the group reviewed the aggregated responses to the questionnaires. If further clarification or elaboration on any statements was required, the questionnaire was adapted and sent back to members with feedback.

## Results

A total of 26 CSs, comprising 62 individual statements, which all reached consensus after multiple rounds of Delphi, were formulated. Two individual statements including “history of atopy as an essential part of penicillin allergy testing” and “systemic immunosuppressants should be withheld at least 4 weeks prior to PST did not reach consensus” did not reach consensus. A summary of the finalized CSs is presented in Table 1. Detailed results of individual response weighting scores are as follows.

**Table 1:** Summary of consensus recommendations for penicillin allergy testing by nonallergists.

**Risk assessment**	
1	The following are essential parts of a penicillin allergy history: a. Duration since index reaction b. Onset time of manifestations after penicillin exposure c. Description of any suspected allergic manifestations after penicillin exposure d. Last exposure to penicillin and reactions (if any) e. Underlying medical conditions/comorbidities f. History of chronic urticaria (>6 weeks in duration)
2	Exclusion criteria for LR allergy testing should include: a. Pregnancy b. Immunocompromised patient (or on systemic immunosuppression in past 4 weeks) c. Active or uncontrolled chronic urticaria d. Unable to withhold medications potentially interfering with skin testing (e.g. anti-histamines, tricyclic antidepressants)
3	Patients with LR features of suspected penicillin allergy can proceed with penicillin allergy testing by a non-allergist.
4	LR features of suspected penicillin allergy should include: a. Unknown or forgotten/untraceable history and event > 1 year ago b. Family history of penicillin allergy only c. Previously told allergy test positive, but no history of reaction d. Other non-*β*-lactam allergies only e. Isolated gastrointestinal upset f. Nonspecific (non-immunological) complaints g. History of non-urticarial rash
5	Patients with any NLR features of suspected penicillin allergy should be referred for evaluation by an allergist
6	NLR features of suspected penicillin allergy should include history of the following after penicillin exposure: a. Anaphylaxis b. Symptoms suggestive of hypotension c. Respiratory compromise d. Urticaria or angioedema e. Documented severe cutaneous adverse reactions f. Mucosal involvement g. Eosinophilia h. Internal organ involvement i. Drug induced autoimmune disease or vasculitis
**Skin Testing**	
7	Skin testing should be performed prior to drug provocation testing
8	Skin testing should be performed at least 8 weeks after (and as soon as possible) following history of suspected allergic reaction after penicillin exposure
9	Antihistamines and tricyclic antidepressants should be withheld at least 1 week prior to skin testing
10	Regarding drug dilutions and reagents: a. SPT followed by IDT at the highest non irritating concentration should be performed b. All SPT should be accompanied by a positive and negative control c. All IDT should be accompanied by a negative control d. SPT and IDT should be performed using recommended concentrations of benzylpenicilloyl-poly-L-lysine, minor determinant mixture, benzylpenicillin and amoxicillin
11	Regarding skin test interpretation: a. SPT is considered positive if a wheal size diameter at least 3 mm larger than negative control, with surrounding erythema b. IDT is considered positive if diameter of the wheal is at least 3 mm greater that the initial wheal, with surrounding erythema c. Delayed IDT readings at 48 to 72 hours may be considered if a non-immediate type reaction is suspected d. Patients with positive SPT or IDT results should be referred for specialist review
**Drug Provocation Testing**	
12	DPT is the gold standard to diagnose genuine penicillin allergy or tolerance
13	DPT should generally be performed when there is a low pre-test probability following negative skin testing
14	DPT should be performed in an appropriate setting with resuscitation facilities readily accessible and under supervision of trained personnel
15	Antihistamines and medications potentially interfering the assessment should be stopped for 7 days before DPT
16	Uncontrolled asthma, active urticaria or other underlying diseases limiting use of rescue medications are relative contraindications for DPT
17	Regarding DPT dosing protocols: a. A 3-step approach (e.g. 10%, 30%, 60% of maximum single unit dose) in 30 minute intervals is recommended b. The index penicillin should be used for DPT (if known) c. If the index penicillin is unknown, DPT should be performed with amoxicillin d. Patient should be observed of at least 1 hour after final dose of DPT
18	An immediate-type hypersensitivity to the DPT agent is confidently excluded if there is no reaction after >1 hour after completion of DPT
19	Patients should be called back at least 72 hours later to ensure there were no non-immediate type manifestations
20	A DPT is considered negative if there is no reaction after at least 72 hours after completion of DPT
21	Patients with reported reactions after DPT should be called back for review and treated as necessary
22	Patients with reported reactions after DPT should referred for specialist review
**Post-testing management**	
23	Inaccurate penicillin allergy labels should be delabelled following a negative DPT and with proper patient counselling
24	Requirement of patient counselling should include: a. Proper patient counselling after both positive and negative workup b. After negative workup, the risk of penicillin allergy is similar to subjects without known allergic history, however, this does not exclude possibility of new sensitization in subsequent years c. After negative workup, penicillin can be prescribed as for usual non allergic subjects
25	After negative DPT, medical records should be updated by: a. Medical records should be properly updated with results of DPT including: DPT agent, dose and date of DPT b. Patients should be given updated physical allergy cards/alerts or alerts or medical alert jewellery
26	Positive skin test or DPT results should be clearly documented in medical records

IDT, intradermal test; LR, low risk; NLR, non-low risk; SPT, skin prick test; DPT, drug provocation testing.

### Risk assessment

***CS #1: The following are essential parts of a penicillin allergy history:***

a. ***Duration since index reaction (score: 0.88 ± 0.22)***
b. ***Onset time of manifestations after penicillin exposure (score: 0.96 ± 0.14)***
c. ***Description of any suspected allergic manifestations after penicillin exposure (score: 0.96 ± 0.14)***
d. ***Last exposure to penicillin and reactions (if any) (score: 0.81 ± 0.25)***
e. ***Underlying medical conditions/comorbidities (score: 0.81 ± 0.43)***
f. ***History of chronic urticaria (>6 weeks in duration) (score: 0.62 ± 0.42)***
  Agreement: 100% with CS #1a–d; 92% with CS #1e and f.

***CS #2: Exclusion criteria for LR allergy testing should include:***

a. ***Pregnancy (score: 0.91 ± 0.20)***
b. ***Immunocompromised patient (or on systemic immunosuppression in past 4 weeks) (score: 0.85 ± 0.43)***
c. ***Active or uncontrolled chronic urticaria (score: 0.88 ± 0.22)***
d. ***Unable to withhold medications potentially interfering with skin testing (e.g. anti-histamines, tricyclic antidepressants) (score: 0.88±0.23)***
  Agreement: 100% with CS #2a, c, and d; 92% with CS #2b.

***CS #3: Patients with LR features of suspected penicillin allergy can proceed with penicillin allergy testing by a non-allergist (score: 0.77 ± 0.33)*.**

Agreement: 92%.

***CS #4: LR features of suspected penicillin allergy should include:***

a. ***Unknown or forgotten/untraceable history and event >1 year ago (score: 0.77 ± 0.26)***
b. ***Family history of penicillin allergy only (score: 0.81 ± 0.26)***
c. ***Previously told allergy test positive, but no history of reaction (score:0.73 ± 0.26)***
d. ***Other non-β-lactam allergies only (score: 0.69 ± 0.43)***
e. ***Isolated gastrointestinal upset (score: 0.96 ± 0.14)***
f. ***Nonspecific (non-immunological) complaints (score: 0.88 ± 0.42)***
g. ***History of non-urticarial rash (score: 0.62 ± 0.55)***
  Agreement: 100% with CS #4a–c and e; 92% with CS #4d, f, and g.

***CS #5: Patients with any NLR features of suspected penicillin allergy should be referred for evaluation by an allergist (score: 0.81 ± 0.33)*.**

Agreement: 92%.

***CS #6: NLR features of suspected penicillin allergy should include history of the following after penicillin exposure:***

a. ***Anaphylaxis (score: 0.88 ± 0.42)***
b. ***Symptoms suggestive of hypotension (score: 0.77 ± 0.44)***
c. ***Respiratory compromise (score: 0.88 ± 0.42)***
d. ***Urticaria or angioedema (score: 0.88 ± 0.42)***
e. ***Documented severe cutaneous adverse reactions (score: 0.92 ± 0.14)***
f. ***Mucosal involvement (score: 0.88 ± 0.42)***
g. ***Eosinophilia (score: 0.69 ± 0.48)***
h. ***Internal organ involvement (score: 0.73 ± 0.53)***
i. ***Drug induced autoimmune disease or vasculitis (0.81 ± 0.48)***
  Agreement: 92% with CS #6a–d and g–i; 100% with CS #6e and f.

### Skin testing

**CS #7: *Skin testing should be performed prior to drug provocation testing (score: 0.85 ± 0.24)*.**

Agreement: 100%.

***CS #8: Skin testing should be performed at least 8 weeks after (and as soon as possible) following history of suspected allergic reaction after penicillin exposure (score: 0.88 ± 0.23)*.**

Agreement: 100%.

***CS #9: Antihistamines and tricyclic antidepressants should be withheld at least 1 week prior to skin testing (score: 0.81 ± 0.25)*.**

Agreement: 100%.

***CS #10: Regarding drug dilutions and reagents:***

a. ***Skin prick tests (SPT) followed by intradermal tests (IDT) at the highest non irritating concentration should be performed (score: 0.81 ± 0.25)***
b. ***All SPT should be accompanied by a positive and negative control (score: 0.92 ± 0.19)***
c. ***All IDT should be accompanied by a negative control (score: 0.88 ± 0.30)***
d. ***SPT and IDT should be performed using recommended concentrations of benzylpenicilloyl-poly-L-lysine (PPL), minor determinant mixture (MDM), benzylpenicillin and amoxicillin (score: 0.96 ± 0.14)***
  Agreement: 100% with CS #10a, b, and d; 92% with CS #10c.

***CS #11: Regarding skin test interpretation:***

a. ***SPT is considered positive if a wheal size diameter at least 3 mm larger than negative control, with surrounding erythema (score: 0.88 ± 0.30)***
b. ***IDT is considered positive if diameter of the wheal is at least 3 mm greater that the initial wheal, with surrounding erythema (score: 0.81 ± 0.38)***
c. ***Delayed IDT readings at 48 to 72 hours may be considered if a non-immediate type reaction is suspected (score: 0.81 ± 0.33)***
d. ***Patients with positive SPT or IDT results should be referred for specialist review (score: 0.92 ± 0.19)***
  Agreement: 92% with CS #11a and c; 85% with CS #11b; 100% with CS #11d.

### Drug provocation testing

***CS #12: DPT is the gold standard to diagnose genuine penicillin allergy or tolerance (score: 0.88 ± 0.22)*.**

Agreement: 100%.

***CS #13: DPT should generally be performed when there is a low pre-test probability following negative skin testing (score: 0.85 ± 0.24)*.**

Agreement: 100%.

***CS #14: DPT should be performed in an appropriate setting with resuscitation facilities readily accessible and under supervision of trained personnel (score: 1.00 ± 0)*.**

Agreement: 100%.

***CS #15: Antihistamines and medications potentially interfering the assessment should be stopped for 7 days before DPT (score: 0.88 ± 0.22)*.**

Agreement: 100%.

***CS #16: Uncontrolled asthma, active urticaria or other underlying diseases limiting use of rescue medications are relative contraindications for DPT (score: 0.85 ± 0.32)*.**

Agreement: 92%.

***CS #17: Regarding DPT dosing protocols:***

a. ***A 3-step approach (e.g. 10%, 30%, 60% of maximum single unit dose) in 30 minute intervals is recommended (score: 0.7 ± 0.35)***
b. ***The index penicillin should be used for DPT (if known) (score: 0.79 ± 0.26)***
c. ***If the index penicillin is unknown, DPT should be performed with amoxicillin (score: 0.79 ± 0.26)***
d. ***Patient should be observed of at least 1 hour after final dose of DPT (score: 0.96 ± 0.14)***
  Agreement: 90% with CS #17a; 100% with CS #17b–d.

***CS #18: An immediate-type hypersensitivity to the DPT agent is confidently excluded if there is no reaction after >1 hour after completion of DPT* (score: 0.85 ± 0.24).**

Agreement: 100%.

***CS #19: Patients should be called back at least 72 hours later to ensure there were no non-immediate type manifestations (score: 0.85 ± 0.32)*.**

Agreement: 92%.

***CS #20: A DPT is considered negative if there is no reaction after at least 72 hours after completion of DPT (score: 0.92 ± 0.19).***

Agreement: 100%.

***CS #21: Patients with reported reactions after DPT should be called back for review and treated as necessary (score: 0.85 ± 0.43)*.**

Agreement: 92%.

***CS #22: Patients with reported reactions after DPT should referred for specialist review (score: 0.92 ± 0.19)*.**

Agreement: 100%.

### Post-testing management

***CS #23: Inaccurate penicillin allergy labels should be delabelled following a negative DPT and with proper patient counselling (score: 0.96 ± 0.14)*.**

Agreement: 100%.

***CS #24: Requirement of patient counselling should include:***

a. ***Proper patient counselling after both positive and negative workup (score: 0.92 ± 0.19)***
b. ***After negative workup, the risk of penicillin allergy is similar to subjects without known allergic history, however, this does not exclude possibility of new sensitization in subsequent years (score: 0.92 ± 0.19)***
c. ***After negative workup, penicillin can be prescribed as for usual non allergic subjects (score: 0.92 ± 0.19)***
  Agreement: 100%.

***CS #25: After negative DPT, medical records should be updated by:***

a. ***Medical records should be properly updated with results of DPT including: DPT agent, dose and date of DPT (score: 0.96 ± 0.14)***
b. ***Patients should be given updated physical allergy cards/alerts or alerts or medical alert jewellery (score: 1.00 ± 0.00)***
  Agreement: 100%.

***CS #26: Positive skin test or DPT results should be clearly documented in medical records (score: 1.00 ± 0.00)*.**

Agreement: 100%.

## Discussion

This document serves as a guide for the management of penicillin allergy and the setup of LR allergy clinics run by nonallergists. These CSs reflect the collective agreement from both allergists and nonallergists of HK-DADI.

There is a massive service gap in providing timely penicillin allergy workups in Hong Kong. However, nonallergists can play a crucial role in penicillin allergy testing, especially for LR cases (19, 20). Experience from the HKWC has shown that around 80% of all referrals for suspected penicillin allergy can be risk-stratified as LR *(manuscript in progress)*. Therefore, we recommend that LR penicillin cases can be managed by nonallergists at Spokes, while NLR cases (or those LR with positive allergy testing) can be referred to the Hub for allergist review. The Hub should also provide adequate training and support for all Spokes assessing LR cases.

A comprehensive allergy history remains the cornerstone for proper risk stratification prior to allergological investigations such as skin testing or drug provocation testing (DPT) (5, 21). For example, a history of anaphylaxis and a short duration since the index reaction have been shown to be important predictors of genuine penicillin allergy (7). Most international authorities recommend penicillin skin testing for suspected penicillin allergies prior to DPT (10, 22). Although DPT remains the “gold standard” in diagnosis, several landmark studies have demonstrated a high negative predictive value of up to 98% of penicillin allergy skin testing (23). The importance of retaining MDM in the diagnosis of *β*-lactam allergy should also be highlighted, especially in Hong Kong (9, 24). To reduce the possibility of false positive and negative skin tests, the panel unanimously agrees to avoid testing patients with active or uncontrolled chronic urticaria, patients on certain medications that may affect skin test interpretation, and immunocompromised patients in the setting of LR allergy clinics. Skin tests should also only be performed at least 8 weeks after the index reaction to bypass the refractory or “anergic” period for all *β*-lactam antibiotics (25). Although positive skin tests during the refractory period (within 8 weeks) could be informative, the risk of false negative tests would necessitate repeat testing in most cases with negative tests. This would not be routinely recommended as it would effectively almost double the cost of allergy testing per patient. Skin test concentrations are well validated and should be performed in accordance with the concentrations outlined by the European Network of Drug Allergy, European Academy of Allergy and Clinical Immunology Interest Group on Drug Allergy, or BSACI (10, 26).

Despite their overall high negative predictive values, there is still a risk of false negative skin testing and, therefore, DPT still remains essential to exclude genuine drug allergy confidently (27). DPT protocols depend on the severity of index reaction and the expertise available in different centers. Although one-step DPT has been demonstrated to be safe, the HK-DADI group agreed that a graded DPT should be generally recommended in the setting of LR allergy clinics (26). The group also agreed that a DPT should only be considered negative if there is no reaction at least 72 h after completion with 100% agreement. However, we recognize that some reactions may take longer to appear depending on the drug dosage used and the index reaction of the drug (26). We acknowledge that there is growing interest in direct DPT testing for low-risk testing (28–30). However, in Hong Kong, drug allergy labels are physician reported, and there is a concern for the safety of direct oral challenge when local data are not yet available. More importantly, there are likely population- and geographical-based differences in penicillin sensitization, and the role of direct DPT in Hong Kong Chinese remains to be elucidated. It is noteworthy that HK has an incidence of only 2% for penicillin allergy (indeed any *β*-lactam allergy) (2). This is incredibly low as most countries report an incidence of 10%–25% (31–33). This figure reflects an accurate point prevalence of *β*-lactam allergies of physician-reported drug allergies in Hong Kong. This discrepancy may be attributed to the inherent differences between inpatients and the general population as well as between ethnicities and regions and a lack of sampling bias. Proper triaging of LR patients by each respective Spoke as determined by these CSs will guide what threshold or DPT strategy we should adopt for LR or NLR cases in the future in our locality. Additionally, if triaging is successful and can be reflected by the high negative predictive value of skin tests, we may shift our practice to direct DPT in the future. Arguably, the most important step after a penicillin workup is proper documentation and counseling (34, 35). Efforts should be made to ensure that patients are educated on the implications after allergy testing with their medical records and drug allergy alert appropriately updated. Inadequate counseling or documentation may lead to continuous unnecessary penicillin avoidance and, therefore, clear written documentation regarding the outcome implications should be provided.

We emphasize that these CSs are by no means definitive and have been designed as a primer and reference for nonallergists. We hope that these CSs can facilitate the integration of a multidisciplinary approach toward tackling the penicillin “allergy” pandemic. We hope that following the prompt implementation of HK-DADI, more data can be generated to refine more specific recommendations in the future. Furthermore, we hope that these CSs can also serve as the foundation for further collaborations and expansion of Immunology and Allergy services in the future.

## Data Availability

The original contributions presented in the study are included in the article/Supplementary Material; further inquiries can be directed to the corresponding author.
